# Maternal postnatal confinement practices and postpartum depression in Chinese populations: A systematic review

**DOI:** 10.1371/journal.pone.0293667

**Published:** 2023-10-30

**Authors:** Xiao Yang, Mujie Qiu, Yichun Yang, Junlin Yan, Kun Tang

**Affiliations:** 1 Health Policy and Management Department, Bloomberg School of Public Health, Johns Hopkins University, Baltimore, Maryland, United States of America; 2 The First Affiliated Hospital of Shantou University Medical College, Shantou, China; 3 Department of Obstetrics, Longgang District Central Hospital of Shenzhen, Shenzhen, China; 4 Vanke School of Public Health, Tsinghua University, Beijing, China; Fondazione Policlinico Universitario Agostino Gemelli IRCCS, Universita’ Cattolica del Sacro Cuore, ITALY

## Abstract

**Background:**

The postpartum period is critical for maternal health status after childbirth. The traditional Chinese postpartum confinement practice, “doing-the-month”, is considered especially effective in helping mothers recover during the postpartum period. However, research has not provided evidence to confirm its benefits. Postpartum depression is a common postpartum disease that seriously threatens maternal health. The systematic review aims to explore the association between “doing-the-month” and postpartum depression in the Chinese female population and to provide a scientific foundation for evidence-based postpartum maternal care.

**Methods:**

Five databases (PubMed, Embase, Web of Science, Scopus, Cochrane, PsycINFO, and Web of Science) were searched according to the protocol (INPALSY202320102). The JBI assessment tool was used to assess the quality of the included studies.

**Results:**

Sixteen quantitative studies from China and Chinese female immigrants in other countries, including 15 cross-sectional studies and 1 randomized controlled study, were identified. Four studies indicated that “doing-the-month” rituals reduced postpartum depression risk while 2 studies showed opposite results; 10 studies did not show a significant association between “doing-the-month” practices and postpartum depression.

**Conclusion:**

There is conflicting evidence regarding the association between “doing-the-month” and the likelihood of developing postpartum depression. Some studies have explored the impact of family ties, particular rituals, and specific stressors during the postpartum period on the occurrence of postpartum depression in Chinese women. According to current research, “doing-the-month” practice failed to show a significant protective effect on postpartum depression in the Chinese maternal population. Evidence-based medical health education for the Chinese postpartum female community is urgently needed.

## Introduction

The postnatal period, also known as the “postpartum period” or “puerperium”, is a decisive period that determines the postpartum health status of the woman [1, 2]. The length of the postpartum period is defined by different research literature as a minimum of 6 weeks to a maximum of 12 months [3–5]. During the postpartum period, the maternal body undergoes a series of changes, both reproductive and systemic, and postpartum morbidity and mortality also mainly occur during this period [6, 7]. Therefore, appropriate care during the postpartum period is critical for maternal and infant health and reducing maternal and infant mortality [1, 8, 9].

The American College of Obstetricians and Gynecologists (ACOG) states that postpartum care shall be an uninterrupted process and emphasizes that mothers should receive regular patient-centered, specialized and comprehensive examinations by health care providers regardless of the time after delivery [10]. The UK NICE guidelines suggest that at least 2 postpartum examinations shall be performed by health workers within 6–8 weeks after delivery [11]. In the guidelines of these developed countries, postpartum confinement is not recommended. In contrast, these high-income countries encourage proper physical activities in the postpartum period to help the mother’s physical health recover more efficiently [12–17]. A balanced diet is also recommended to new mothers by official guidelines including *The Dietary Guidelines for Americans* and *NHS Breastfeeding and Diet* [18, 19].

Many low- and middle-income countries in East Asia, Southeast Asia, the Middle East, Latin America, and Africa follow local traditions for postpartum care after childbirth, such as restricting maternal activities, limiting diet, staying warm, and relying on family members, among other traditional practices [20–31].

In the Chinese maternal population, traditional postpartum care is known as "doing-the- month" (DTM) or “zuoyuezi”, and it lasts from childbirth to one month after delivery [24]. DTM is originated from traditional Chinese medicine (TCM), which can be dated back to 2,000 years ago, with the "Yin and Yang" theory serving as the guiding principle [32]. According to the theory of TCM, the entire pregnancy and childbirth process consumes women’s "Yang" (representing heat, warmth, and sunshine) while producing more "Yin" (referring to cold, dark), resulting in an imbalance of women’s health status [33]. Meanwhile, the DTM process is considered critical in restoring the mother’s Yin and Yang balance. Specifically, the behaviors that need to be performed during DTM include restricting activities, especially prohibiting women from leaving the bed; prohibiting eating raw or cold foods; reducing personal cleaning procedures, such as bathing or washing hair, to a minimum extent; avoiding catching cold by not opening windows for ventilation; and prohibiting sex [20, 33, 34]. According to studies, the prevalence of DTM among the Chinese maternal population is as high as 90%, and most Chinese women adopt one of the required behaviors of DTM after delivery [34, 35].

Although DTM has traditionally been thought to help mothers recover better and is still practiced by many Chinese women in modern times, there is very little evidence to support the benefits of DTM. *Chien et al*. reported that higher adherence to DTM is associated with lower PPD scores and less severe physical symptoms [34]. A qualitative study by *Raven et al*. stated that DTM is beneficial to maternal health as reported by family members or health workers [36]. *Liu et al*. found that the fatigue level reported by Chinese women who practiced DTM was lower than that reported by women from other cultures, which may be attributed to sufficient support from the family [37]. However, many DTM habits can lead to adverse health consequences in the general population. For example, restricted physical activities can cause muscle disuse atrophy [38, 39], musculoskeletal pain [40–42], and decreased cardiorespiratory function [43, 44]; in addition, a less active lifestyle is also associated with higher risks of developing chronic diseases such as type 2 diabetes [45, 46]. Reduced sunshine exposure due to a lack of outdoor exercise may contribute to vitamin D deficiency [47, 48], sleep disturbance and depression [49–52]. Inadequate personal hygiene may result in the development of systemic or localized infections [53, 54]. Moreover, unbalanced diets (a lack of fruits and vegetables and a high fat intake) also lead to deficiencies in essential nutrients or increase the possibility of obesity [55–58].

Due to the unique nature of the postpartum period as the “fourth trimester” that the maternal body undergoes significant physiological changes and emotional adjustments, mothers may be particularly vulnerable to these traditional confinement practices as they restrict women’s normal activities. These actions, which are conducted without the support of evidence-based medicine, may affect mothers both physically and mentally in both the short and long term.

Postpartum depression (PPD) is one of the most common complications in the postpartum period, affecting 10%-15% of postpartum women on average [59]. Data from different countries show that the PPD prevalence can reach up to 60% [60]. The main clinical manifestations of the disorder include depressed mood, sleep disturbances, anhedonia, psychomotor disorders, feelings of guilt or hopelessness, low self-esteem, and severe PPD can lead to suicidal behaviors [61]. Major risk factors for PPD include previous history of depression or other psychiatric disorders, high-risk pregnancy, stressful events and lack of social support during the postpartum period, and fluctuations in hormone levels [61, 62]. On the other hand, certain lifestyle factors, appropriate physical activity, adequate sleep, and vitamin supplementation usually protect mothers from PPD, whereas those women who embraced DTM practices may be deficient in these beneficial behaviors [63–65].

PPD affects the well-being of mothers and babies, and the specific lifestyles such as physical inactivity and inadequate dietary intake that may lead to the development of PPD are in concordance with the traditional practices of DTM. Therefore, the purposes of this review are to investigate the association between traditional postpartum confinement practices and PPD in Chinese women and to provide scientific evidence for maternal health recovery practices during the postpartum period (INPLASY202320102).

## Methods

A protocol has been developed for the systematic review (INPALSY202320102). A literature search was conducted in 5 databases, including PubMed, Embase, Web of Science, Scopus, Cochrane, PsycINFO and Web of Science, between 2000 and 2022 according to the Preferred Reporting Items for Systematic Reviews and Meta-Analysis (PRISMA) statement [66]. Studies were included if they were published in English and included female human participants. Free text used for the search were as follows: “traditional postnatal practice” or “postnatal confinement” or “postnatal tradition” or “postnatal ritual” or “traditional postpartum practice” or “postpartum confinement” or “postpartum tradition” or “postpartum ritual” or “doing-the-month” or “zuo-yue-zi”, and “depression”, and “Chinese” or “China”. Medical subject headings (MeSH) terms related to depression were also applied during the search. In vivo, in vitro, in silico and animal studies were excluded, as were meta-analyses, reviews, qualitative studies, case reports, and studies with only abstracts or protocols available.

The database search was conducted independently by two reviewers, X.Y and M.Q. Then, the two reviewers independently screened the article titles for further assessment, assessed the abstracts to determine potential inclusion, conducted comprehensive reviews of the selected articles, and made final selection for the systematic review. Additionally, a manual cross-reference search was carried out based on the selected articles. At each step of the process, a third author (K.T.) was invited for discussion and decision if there were inconsistent opinions between the two reviewers.

Once a specific study was selected for inclusion, the following data were extracted: the author information, year of publication, country of research conducted, type of study, study population, sample size, definition of the postpartum period, study duration, assessment instrument for depression, study outcome and strength of the association.

Following final inclusion, each article was evaluated for its quality. The JBI critical appraisal tool was implemented to evaluate the cross-sectional studies and randomized controlled trials that were selected [67]. Cross-sectional studies were examined with 8 questions and a maximum score of 16 points (high quality: 12–16 points, moderate quality: 7–11 points, low quality: ≤6 points); randomized controlled studies were rated with 13 questions and a maximum score of 26 points (high quality: 18–26 points, moderate quality: 10–17 points, low quality: ≤9 points). Table 1 shows the results of the quality evaluation of the included studies. The outcomes of the included studies were categorized into 3 domains regarding the effect of DTM on PPD, namely “Yes”, “No” or “Inconclusive”.

**Table 1:** Quality assessment of the included studies.

Quality assessment for included cross-sectional studies										
First author & Year of publication	Criteria for inclusion	Description of study subjects & setting	Measurement of exposure	Measurement of condition	Identification of confounders	Method to control confounders	Measurement of outcomes	Use of statistical analysis	Overall score	Quality rating
SSK Leung, 2001 [68]	2	2	2	2	0	0	2	2	12	Moderate
Matthey *et al*., 2002 [35]	2	2	1	2	0	0	2	0	9	Moderate
Heh *et al*., 2004 [69]	2	2	0	2	0	0	2	1	9	Moderate
Chien *et al*. 2006 [34]	2	2	1	2	2	2	2	2	15	High
Wan *et al*., 2008 [70]	2	2	1	2	1	2	2	2	14	High
Hung *et al*., 2010 [71]	2	2	0	2	1	2	2	2	13	High
Chen *et al*., 2012 [72]	2	2	2	2	2	2	2	2	16	High
Liu *et al*., 2012 [37]	2	1	2	2	0	0	2	2	11	Moderate
Chang *et al*., 2014 [73]	2	2	1	2	1	2	2	2	14	High
Ho *et al*., 2015 [74]	2	2	2	2	2	2	2	2	16	High
Teo *et al*., 2018 [75]	2	2	2	2	2	2	2	2	16	High
Ding *et al*., 2020 [76]	2	2	2	2	2	2	2	2	16	High
Guo *et al*., 2021 [77]	2	2	2	2	2	2	2	2	16	High
Peng *et al*., 2021 [78]	0	0	1	2	1	2	2	2	10	Moderate
Zhao *et al*., 2022 [79]	2	2	2	2	2	2	2	2	16	High

Quality assessment for the included randomized control trial															
First author & Year of publication	True randomi-zation	Allocation concealment	Baseline characteristics	Blind to participants	Blind to deliverers	Blind to assessors	Identically treated	Adequate follow-up	Analyze of participants	Identical measure-ment of outcomes	Reliability of outcome measures	Use of statistical analysis	Trial design	Overall score	Quality rating
Liu *et al*., 2020 [80]	2	2	2	0	2	1	2	2	2	2	2	1	2	22	High

## Results

### Selection of studies

**Fig 1:**
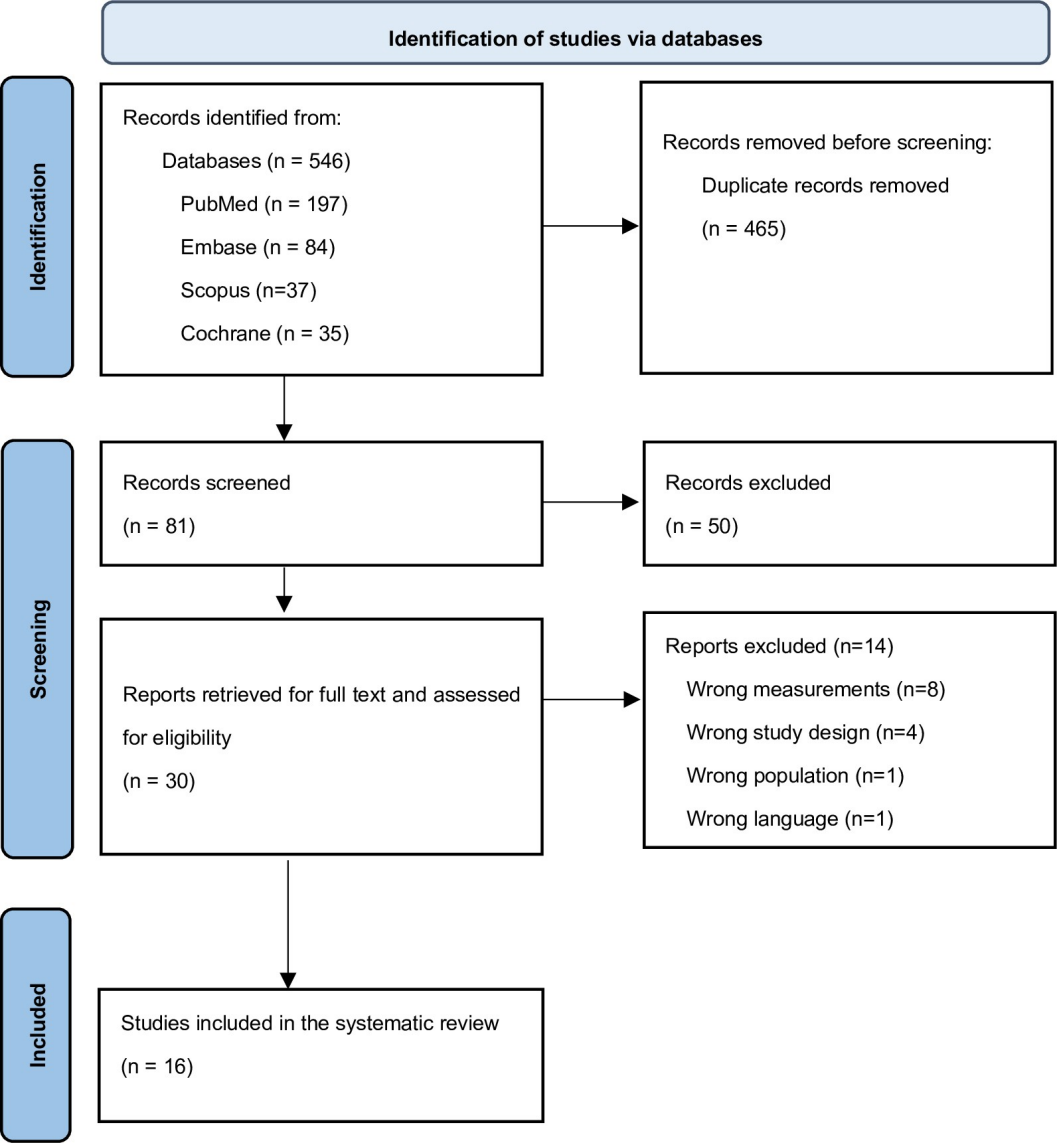
PRISMA flow diagram of study inclusion.

A total of 546 publications were initially identified from the databases (PubMed, n = 197; EMBASE, n = 84; Scopus, n = 37; Cochrane, n = 35; PsycINFO, n = 30; Web of Science, n = 163). A total of 465 articles were identified as duplicates and were removed. The remaining 81 titles and abstracts were screened, and 30 full-text articles were retrieved. Fourteen papers were excluded because they did not measure DTM or PPD (n = 8); had a qualitative study design (n = 4); had a population that was not Chinese or from China (n = 1); or the language was not English (n = 1). Sixteen publications were included for final review. The PRISMA flow diagram is used to demonstrate the study selection process (Fig 1). The studies included in the review are listed in Table 2.

**Table 2:** Studies investigating the association between traditional Chinese “doing-the-month” practices and postnatal depression.

First author & year of publication	Study location	Characteristics of the study population	Study design	Data collection timepoint(s)	Instruments for PPD and/or mental health status	Prevalence/adherence of traditional postpartum practices	Key findings	Protective effects of DTM regarding PPD
SSK Leung, 2001[68]	Hong Kong, China	385 women from 5 HA hospitals	Cross-sectional design within a longitudinal study	6 weeks and 6 months postpartum	EPDS, Perceived Stress Scale (PSS), Childcare Stress Inventory (CSI), Postpartum Support Questionnaire (PSQ), Postpartum Social Support Questionnaire (PSSQ)	70.9% (n = 273) of the interviewed women followed DTM	Of the 76% women who had someone to help them for DTM, 22% showed depressive symptoms, which was 7.5% higher than those who did not have someone to help during the DTM period.	Inconclusive
Matthey *et al*., 2002 [35]	Sydney, Australia	102 Chinese female immigrants in Sydney, Australia	Cross-sectional study	6 weeks postpartum	Edinburgh Postnatal Depression Scale (EPDS) and General Health Questionnaire (GHQ)	90.2% of the 102 participants followed cultural practices	There was no difference in the EPDS score between those who did not follow culture practices and those who followed cultural practices. The GHQ score showed that those who followed cultural practices had a greater level of stress than the women who did not follow the practice (p<0.001).	Inconclusive
Heh *et al*., 2004 [69]	Taiwan, China	240 women attending 2 teaching hospitals in Taipei	Cross-sectional study	N.A.	PSSQ & EPDS	100% *	Of the 186 respondents, 78 (42%) experienced depressive symptoms and 21% had an EPDS score≥10. Living with the parents-in-law at their home and having the mother-in-law as the main helper during DTM were significantly associated with higher EPDS scores (p<0.01).	Inconclusive
Chien *et al*., 2006 [34]	Taiwan, China	202 women from 2 hospitals and 1 postnatal care center	Cross-sectional study	4–6 weeks postpartum	The Chinese version of the Center for Epidemiologic Studies Depression Scale (CES-D)	Mean adherence to DTM practices was 75.7 (SD = 14.5; range = 33–108)	Those who had higher scores of depressions tended to have lower adherence to DTM practices, but the trend was not statistically significant (p = 0.11). Adherence to DTM practices decreased the odds of depression (OR = 0.97, 95% CI 0.95–0.997, p = 0.03). Women who practiced DTM in postpartum care centers or while staying with their friends or relatives had lower odds of depression compared to those who practiced DTM at their own homes.	Yes
Wan *et al*., 2008 [70]	Mainland China	342 women from the obstetric outpatient clinic in an academic medical center in Beijing	Cross-sectional study	6–8 weeks postpartum	EPDS	96% (n = 327) practiced “Zuo-yue-zi”	The practice of “Zuo-yue-zi” was not associated with PPD (OR 0.92; 95% CI, 0.20–4.25). Women whose caregiver was their mother-in-law (35.8%, OR = 2.06, 95% CI 1.08–3.92) and women who believed that zuoyuezi was not helpful (45.1%, OR = 2.26, 95% CI 1.21–4.19) had twice the odds of PPD.	Inconclusive
Hung *et al*., 2010 [71]	Taiwan, China	258 women stayed in one of eight postpartum nursing centers in Kaohsiung, Taiwan for at least 20 days	Cross-sectional study	After discharge from the nursing center	The Hung Postpartum Stress Scale (Hung PSS), the Social Support Scale (SSS) and the 12-item Chinese Health Questionnaire (CHQ)	All of the 98.1% respondents	Women who did not have psychiatric morbidities were found to have significantly longer lengths-of-stay than women that had minor psychiatric morbidity (p = 0.047). Top postpartum stressors including the women’s negative self-perceptions about their body shape after childbirth, sleep disturbances and the unpredictability of the baby’s needs. Tso-Yueh-Tzu as practised in postpartum nursing centres gave the postpartum women the opportunity to receive tangible support and, therefore, helped decrease postpartum stress and improved their general health.	Inconclusive
Chen *et al*., 2012 [72]	Taiwan, China	190 participants (Chinese, n = 109; Vietnamese, n = 81)	Cross-sectional study	N.A.	EPDS	The mean adherence to DTM was 79.9% (86.25±11.86) among Chinese immigrants	Adherence to DTM practices was significantly negatively associated with PPD symptoms among Chinese immigrants to Taiwan (p<0.001). Multiple logistic regression indicated that adherence to DTM practices significantly negatively associated with PPD (OR = 0.93, 95% CI 0.90–0.96).	Yes
Liu *et al*., 2012 [37]	Mainland China	198 women from a postpartum unit of an urban maternity hospital in Hubei, China,	Cross-sectional study	6 weeks postpartum	EPDS	The mean adherence to DTM was 69.6 (SD = 10.5, R = 31–99)	PPD was significantly positively correlated with adherence to DTM (p<0.05).	No
Chang *et al*., 2014 [73]	Taiwan, China	213 women (102 mothers of preterm infants and 111 mothers of full-term infants)	Cross-sectional study	6–48 months after childbirth	CES-D, the Maudsley Personality Inventory, Family Apgar Index	93.1% (n = 95) of the mothers of preterm infants and 95.5% (n = 106) of the mothers of the full-term infants followed postpartum confinement practices	Lack of postpartum confinement was associated with more depressive symptoms (OR = 4.01, 95% CI = 1.22–13.19).	Yes
Ho *et al*., 2015 [74]	Taiwan, China	341 women at the obstetric outpatient clinic of China Medical University Hospital, Taiwan, China	Cross-sectional study	4–6 weeks after delivery	Beck’s Depression Inventory (BDI II), EPDS, Beck Anxiety Inventory (BAI), the Pittsburgh Sleep Quality Index (PSQI)	Adherence to restricted behaviours in DTM varied from 3.5% to 62.8%	After adjusted for confounding factors, bathing or showering was an independent indicator for low BDI II and EPDS scores (p<0.05). In contrast, touching cold water was an independent indicator for high BDI II scores, while squatting was an independent indicator for high BDI II and EPDS scores (p<0.05).	Inconclusive
Teo *et al*., 2018 [75]	Singapore	490 participants from the GUSTO study, in which 312 were Chinese ethnicity	Cross-sectional design within a prospective study	26–28 weeks at gestation and 3 weeks post delivery	EPDS, State-Trait Anxiety Inventory (STAI)	The dietary pattern scores of the Traditional Chinese Confinement diet ranged from 0.34 to 0.70, with higher scores indicating higher adherence to the dietary pattern	There was no association observed between Traditional-Chinese-Confinement diets and postpartum mental health (OR-1.3, 95% CI 0.85–2.00, p = 0.22).	Inconclusive
Ding *et al*., 2020 [76]	Mainland China	2615 women identified from Shanghai Birth Cohort	Cross-sectional study	42 days postpartum	EPDS	60.5% of the participants strictly followed the traditional confinement practice	After adjusted for confounding factors, women who went outside their home showed higher risks of PPD compared to their confined peers, and a dose‒response relationship of the frequency of going out and PPD was observed (1–2 times: OR = 1.9, 95% CI = 1.4–2.4; 3–5 times: OR = 2.3, 95% CI = 1.5–3.5; 6 times: OR = 2.5, 95% CI = 1.2–5.1). The practice of window-opening was associated with reduced risks of PPD compared to those who do not open or sometimes open the window (often: OR = 0.6, 95% CI = 0.4–0.9; always: OR = 0.4, 95% CI = 0.3–0.7). Hair washing 1–2 times during DTM is associated with a higher risk of PPD compared to those who never washed their hair during DTM (OR = 1.5, 95% CI = 1.1–2.2).	Inconclusive
Liu *et al*., 2020 [80]	Mainland China	124 women (experimental group with antenatal health education, n = 62; control group, n = 62) at a community health center in Wuhan, China	Single-blinded, randomized controlled trial	1 week and 4 weeks postpartum	EPDS, postpartum symptom checklist (PSC)	After intervention, the mean adherence to DTM (ADP) score of the experimental group was significantly lower than that of the control group (p<0.001).	The control group had a higher percentage of participants (20.4%) with increased EPDS scores (≥10) compared with that in the experimental group (14.8%). However, there was no statistically significant difference in terms of the mean PSC or EPDS between the intervention group and control group at 4 weeks postpartum, nor between weeks 1 and 4 after delivery for the intervention group or for the control group (p>0.05).	Inconclusive
Guo *et al*., 2021 [77]	Mainland China	955 women from urban and rural healthcare facilities in Hunan, China	Cross-sectional study	4–10 weeks after delivery	EPDS	Adherence to DTM varied between different practices, with the highest adherence to not having sexual intercourse (98.1%, n = 937) and the lowest adherence to not brushing teeth (10.2%, 97/955)	Moderate and low adherence to postpartum practices were associated with higher EPDS scores (p<0.001): Moderate adherence: adjusted difference 1.07, 95% CI 0.20, 1.94 Low adherence: adjusted difference 1.72, 95% CI 0.84, 2.60 Lower postpartum practice satisfaction was associated with higher risks of PPD symptoms (p<0.001).	Yes
Peng *et al*., 2021 [78]	Mainland China	1,325 women from Shenzhen Maternity and Child Health Hospital	Cross-sectional study	14–60 days postpartum	EPDS	100% *	26.5% of the participants had PPD. No association was found between different caregivers and the risk of developing PPD during DTM, (p>0.05): Care provided by yuesao, OR = 1.203, 95% CI 0.949–1.643 Care provided by mother-in-law, OR = 1.249, 95% CI 0.949–1.643 Care provided by other relatives, OR = 0.949, 95% CI = 0.579–1.554.	Inconclusive
Zhao *et al*., 2022 [79]	Mainland China	955 women from the YI study which recruited participants from 10 cities in China	Cross-sectional study	Within 365 days postpartum	EPDS	59.5% of participants adhered to certain postpartum practices; 36.5% of participants had dietary-related postpartum practices	Adherence to overall postpartum practices and dietary practices were associated with a higher risk of PPD (overall postpartum practices, OR = 1,44, 95% CI 1.08–1.92, p = 0.012; dietary practices, OR = 1.43, 95% CI 1.07–1.92, p = 0.016). Compared with those who did not adhere to postpartum practices, women who experienced the practices had a significant PPD-related dietary pattern loading (high intake of meat and eggs and a low intake of vegetables, mushrooms, and nuts).	No

Abbreviations: DTM: “doing-the-month”; PPD: postnatal depression; HA: hospital authority; N.A: not applicable; EPDS: Edinburgh Postnatal Depression Scale; OR: odds ratio; CI: confidence interval.

* The study only included those who followed DTM practices; therefore, the prevalence was calculated as 100%.

### Characteristics of the included studies

Of the 16 included studies, the majority were conducted in Mainland China (n = 7) or Taiwan, China (n = 6); the remaining 3 studies were conducted in Hong Kong China, Sydney and Singapore. Fifteen studies adopted a cross-sectional study design, 2 of which were embedded in a longitudinal study or extracted data from a prospective study database, while one study used a randomized controlled study design. In most studies (n = 12), PPD was measured during or immediately after the confinement period; in two of the remaining studies, the time interval between delivery and measurement of PPD was one and four years postpartum, respectively, and another 2 studies did not specify the time PPD was measured in the manuscripts.

### Instruments used for PPD measurement

The Edinburgh Postnatal Depression Scale (EPDS) was the most commonly used measurement tool and was adopted by thirteen studies, as it is an instrument specifically developed for PPD [81]. Most studies (n = 12) employed a Chinese-translated version of the EPDS scale because the participants were Chinese populations or Chinese immigrants [82]. Six out of the thirteen studies used ≥10 as the cutoff point to indicate probable PPD among Chinese women [82]; three studies indicate 12/13 as the cutoff scores for PPD, the threshold used in the original EPDS [81]; and two studies adopted a 2-tier EPDS scoring approach (first-tier: ≥9 or ≥10; second tier: ≥13) to identify different severities of depression. In the other two publications, researchers did not report a clear cutoff of EPDS.

In addition to the EPDS, other scales included the Hung scale developed by a Taiwanese researcher, which is also an instrument specifically used to measure PPD (n = 1) [83], and the Chinese version of the Center for Epidemiological Studies-Depression scale (CES-D, n = 2) [84].

### Studies showed protective effects of DTM against PPD

Among all included studies, 4 studies (25%) showed beneficial implications of the traditional Chinese DTM practice on PPD symptoms for Chinese women, of which 3 studies were conducted in Taiwan and 1 in mainland China. *Chien et al*. investigated the relationship between compliance with traditional postpartum confinement rituals and PPD and discovered that Taiwanese women with poor confinement compliance had a higher trend (p = 0.11) of PPD. The study also found that when a mother is confined in other familiar households or in a specialized postpartum rehabilitation center during the DTM period, her chances of developing PPD are significantly lower than when she is confined in her own home (p<0.01) [34]. Another study conducted in Taiwan by *Chen et al*. yielded similar results: after controlling for potential confounding variables, cognitive recognition and compliance with DTM customs were significantly inversely correlated with depression status (p<0.05) [72]. *Chang et al*. recruited mothers with preterm and full-term pregnancies in Taiwan and found a PPD rate of approximately 28% among all mothers, with the child-bearer of preterm infants having a 3% higher rate of depression than mothers with full-term offspring. More importantly, mothers who were not confined postpartum had a higher OR of PPD than those who complied with postpartum confinement in both preterm and full-term pregnancies, with 3.5-fold and 4.6-fold ORs, respectively [73]. A study from Mainland China investigated the effect of adherence and satisfaction with DTM on PPD in nearly 1,000 women and found that a trend toward lower satisfaction with DTM was significantly associated with the development of PPD [77]. From the perspective of adherence to DTM practices, low adherence to restrictions on housework and social activities was significantly associated with an increasing trend in PPD (p<0.05), while low to moderate DTM adherence was significantly associated with an increasing trend in EPDS scores (p<0.05) [77].

### Studies showed adverse effects of DTM on PPD

A total of 2 studies indicated a negative association between traditional Chinese postpartum practices and the mental health status of Chinese women. *Liu et al*. examined the influence of adherence to DTM on physical and psychological health among 198 women in Hubei Province, China, by a repeated descriptive study at 3 days and 6 weeks postpartum, which revealed that the maternal depression status in all participants significantly worsened at 6 weeks postpartum compared to the immediate postpartum timepoint (p<0.001). In addition, women who were more obedient to the rules of DTM were more prone to develop PPD (p<0.05) [37]. A recent study published by *Zhao et al*. investigated women in 10 cities in China and found that not only was the risk of depression nearly 40% significantly higher among women with high compliance to DTM practice, but specifically, the risk of PPD increased by approximately 30% among mothers who followed certain DTM recommended recipes [79].

### Studies that are inconclusive on the effects of DTM on PPD

In most of the publications included in this review, no significant associations were demonstrated regarding the impact of Chinese traditional postpartum practice on the risk of developing PPD. Although no statistically significant relationship was found between adherence to DTM rituals and developing PPD, a randomized controlled trial conducted by *Liu et al*. observed a trend of lower adherence to DTM and a relatively lower risk of depression among mothers who had prenatal health education [80]. Moreover, several studies have demonstrated the influence of family relationships and housing situations during the postpartum period on the risk of PPD. Studies by *Heh et al*. and *Wan et al*. revealed a higher probability of PPD among mothers who were living in their in-laws’ residence or who had their in-laws as the major care-takers during the DTM period [69, 70]. Interestingly, Leung’s study of postpartum women in Hong Kong found a higher risk of depression among women who had external support from a caregiver than women who did not have a caregiver during the DTM [68]. *Peng et al*. did not find a significant association between different care takers during DTM for Chinese mothers and the development of PPD [78].

Adherence to specific behaviors of DTM has also been assumed to be associated with the increased or decreased risk of PPD, but evidence from different studies was conflicting. For example, it was found that bathing or showering was significantly associated with a reduced risk of PPD [74], but occasional hair washing or touching low-temperature water during the postpartum period significantly increased the risk of depression [74, 76]. Ventilation through window opening reduced women’s risk of PPD [76]; however, outdoor activity increased maternal depression risk in a dose-effect manner [76], and squatting exercise was also found to be correlated with a higher likelihood of PPD [74]. Inconsistent with the aforementioned evidence that a DTM diet pattern can significantly elevate the risk of PPD [79], *Teo et al*. found no association between following a DTM diet and the onset of PPD [75].

### Sensitivity analysis stratified by univariate and multivariate adjustments

The included studies were categorized according to the analytical methods employed, either univariate analysis or multivariate analysis, to investigate the potential influence of these methods on the protective effect of DTM against PPD with or without adjust for potential confounders. Four of the 16 included studies used univariate analysis, three of which results were inconclusive and one was negative in terms of the protective effect of DTM on PPD. Eleven studies, on the other hand, performed multivariate analysis to explore the protective impact of DTM on PPD. Among them, the conclusions of 6 studies were inconclusive, 4 concluded that there was a protective effect of DTM against PPD, while the remaining 1 study concluded that there was a negative effect.

## Discussion

This review investigated the relationship between traditional Chinese postpartum practices and the risk of PPD in Chinese women and discovered inconsistencies in the findings of the current evidence. Despite the fact that the majority of research was unable to reach definitive conclusions regarding the effect of DTM on PPD, these studies did explore the impact of intimate relationships, adherence to particular conventional postpartum customs, and specific postpartum stressors on the occurrence of PPD in women.

In this systematic review, we found that most of the included studies suggested no significant association between DTM and PPD. Moreover, in those studies which found significant associations, the findings were somewhat contradictory. Some of these studies suggested a reduced risk for PPD among those who practiced DTM, while other indicated an increased risk for PPD. When analyzing the different components of DTM, there were inconclusive findings. Most of the studies found that limiting physical activity may significantly reduce the risk of postpartum depression, while one study suggested the contrary. The social and family support, which is another important component of DTM, also showed mixed results. Some studies have found that support from society and family when practicing DTM is significantly associated with reduced risk of PPD. However, other studies found that specific caregivers who provided care during the DTM period, such as mothers-in-law, is also significantly associated with increased risk of PPD. Current evidence is still insufficient to draw any definitive conclusion for the association between DTM and PPD, given the complexity of the cultural, psychosocial, and behavioral nature of DTM in the Chinese society.

To our knowledge, this is the first article in the last decade to review the association between adherence to DTM and PPD among Chinese women, which added new evidence for the association between adherence to DTM and risk of PPD. This review includes both female residents of mainland China and populations of women from other countries and regions that follow Chinese culture, making the research evidence more comprehensive. Most of the interviews in the included studies were conducted during or immediately after DTM, and respondents had a high recall accuracy rate.

The main limitations include that this review only selected studies published in English, but the population investigated was Chinese female residents or women living with Chinese cultural traditions, so there is a possibility of missing corresponding evidence in the Chinese language. In addition, this review only included quantitative studies, while some previously published qualitative studies explored the association between individual women’s DTM practices and the risk of developing PPD in more depth by means of interviews [85–88]. The majority of the included studies were cross-sectional, which made it difficult to establish a causal relationship between the exposure of adherence to DTM and the outcome of PPD among Chinese women. Only one study used a randomized controlled study design, and a trend was discovered toward higher rates of PPD in women who adhered to DTM and did not receive prenatal health education compared to those who did, but the difference was not statistically significant, which may be related to maternal nonobedience with certain behaviors, especially confinement, in both groups [80]. Furthermore, all four studies indicating the protective effect of DTM behavior on PPD adopted a multivariate analysis method. Although the total number of included studies is limited and no definitive conclusion can be established, it indicates that future studies should account for confounding factors in order to adequately explain the effect of DTM on PPD. In addition, the majority of the participants included in the available studies were recruited from maternal and child departments or postpartum rehabilitation centers, which may result in selection bias by not including mothers who were unable to deliver in a hospital and may have a relatively low educational and economic level, poorer mental health and a greater belief in traditional DTM practices [89–92] .

A study conducted as early as the 1970s concluded that Chinese traditional postpartum practices, especially postpartum physical restraints, have positive implications for women after giving birth [93]. Although subsequent studies have reached inconsistent conclusions regarding DTM and the risk of PPD, several common factors that have an impact on PPD were still identified by this review. During the DTM period, both the presence of family support and satisfaction with family support may affect the occurrence of PPD. Not only is the existence of support critical, but PPD may also depend on who is the primary caregiver during DTM [34, 69, 70]. This may be due to the influence of different living habits on maternal emotions during the critical stage of the postpartum period. In two studies in Taiwan and mainland China, it was found that the incidence of PPD was significantly higher in women who frequently squatted and went outdoors during DTM, which seems to be in line with the traditional concept that during DTM, physical activities should be reduced as much as possible. However, the deep-seated reason may be that the mother has to do some physical labor because of a lack of adequate support or needs to go out to work due to economic reasons, which could affect her emotions [74, 76]. In addition, although several studies have shown that compliance with DTM practices is associated with a reduced risk of depression, it is more likely to be due to the mother’s receiving sufficient life and mental support rather than restricting maternal activity and eating an unbalanced diet [34, 72, 73, 77].

## Conclusion

In conclusion, the available evidence is insufficient to support the benefit of traditional confinement practice in reducing the risk of PPD. Future research could adopt a prospective study design, cover a wider group of Chinese women and take into account family support, mental impact, and socioeconomic factors during the DTM period. At the same time, when developing perinatal health interventions, consideration should be given to not only educating women and their husbands concerning the importance of appropriate postpartum care but also educating their family members to implement best practices, including moderate activity and a nutritionally balanced diet to ensure the well-being of the mothers.

## Supporting information

S1 Checklist PRISMA checklist for maternal postnatal confinement practices and postpartum depression in Chinese populations: A systematic review. (DOCX)

S1 File INPALSY protocol for maternal postnatal confinement practices and postpartum depression in Chinese populations: A systematic review. (PDF)
