# Supplementary material for: Maternal postnatal confinement practices and postpartum depression in Chinese populations: A systematic review
Source: PLoS One. 2023 Oct 30;18(10):e0293667. doi: 10.1371/journal.pone.0293667 (PMC10615300; doi:10.1371/journal.pone.0293667)
Supplement: S1 File — (PDF) [file pone.0293667.s002.pdf]

International Platform of Registered Systematic Review and Meta-analysis Protocols

INPLASY PROTOCOL

To cite: Yang et al. Maternal Postnatal Confinement Practices and Postpartum Depression in Chinese populations: A Systematic Review. Inplasy protocol 202320102. doi: 10.37766/inplasy2023.2.0102

Received: 22 February 2023

Published: 22 February 2023

Corresponding author:
Kun Tang

tangk@tsinghua.edu.cn

Author Affiliation:
Vanke School of Public Health, Tsinghua University.

Support: **None.**

Review Stage at time of this submission: **Completed but not published.**

Conflicts of interest:
None declared.

Maternal Postnatal Confinement Practices and Postpartum Depression in Chinese populations: A Systematic Review

Yang, X[1]; Qiu, MJ[2]; Yang, YC[3]; Tang, K[4].

Review question / Objective: The postnatal period, also known as “postpartum period” or “puerperium”, is defined as a the time from the moment a pregnant woman gives birth to a period of several weeks to several months thereafter and is a decisive period that determines the postpartum health status of thewoman.In the Chinese maternal population, traditional postpartum care is known as "doing-the-month" (DTM) or “zuoyuezi”, and it lasts from childbirth to one month after delivery.Postnatal depression (PPD) is one of the most common complications in the postpartum period, affecting 10%-15% of postpartum women on average.PPD affects the well-being of mothers and babies, and the specific lifestyles that may lead to the development of postpartum depression is in concordance with the traditional practices of DTM. Therefore, the purpose of this review is to investigate the association between traditional postpartum confinement practices and postpartum depression in Chinese women, and to provide scientific evidence for maternal health recovery practices during the puerperium.

INPLASY registration number: This protocol was registered with the International Platform of Registered Systematic Review and Meta-Analysis Protocols (INPLASY) on 22 February 2023 and was last updated on 22 February 2023 (registration number INPLASY202320102).

INTRODUCTION

Review question / Objective: The postnatal period, also known as “postpartum period” or “puerperium”, is defined as a the time from the moment a pregnant woman gives birth to a period of several weeks to several months thereafter and is a decisive period that determines the postpartum health status of thewoman.In the Chinese maternal population, traditional postpartum care is known as "doing-the-month" (DTM) or “zuoyuezi”, and it lasts from childbirth to one month after delivery.Postnatal depression (PPD) is one of the most common complications in the postpartum period, affecting 10%-15% of postpartum women on average.PPD affects the well-

Yang et al. Inplasy protocol 202320102. doi:10.37766/inplasy2023.2.0102 Downloaded from https://inplasy.com/inplasy-2023-2-0102/

being of mothers and babies, and the specific lifestyles that may lead to the development of postpartum depression is in concordance with the traditional practices of DTM. Therefore, the purpose of this review is to investigate the association between traditional postpartum confinement practices and postpartum depression in Chinese women, and to provide scientific evidence for maternal health recovery practices during the puerperium.

Condition being studied: Postnatal depression (PPD) is one of the most common complications in the postpartum period, affecting 10%-15% of postpartum women on average.59 Data from different countries show that the postpartum depression rate can reach up to 60%.60 The main clinical manifestations of the disorder include depressed mood, sleep disturbances, anhedonia, psycho-motor disorders, feelings of guilt or hopelessness, low self-esteem, and severe postpartum depression can lead to suicidal behaviors.61 Major risk factors for postnatal depression include previous history of depression or other psychiatric disorders, high-risk pregnancy, stressful events and lack of social support during the postpartum period, low breastfeeding rate, fluctuation in hormone levels.61,62 Certain lifestyles, such as lack of adequate physical activity, sleep deprivation, insufficient supplement of specific vitamins and micronutrients, are also important risk factors for postpartum depression.

METHODS

Search strategy: PubMed, Embase, Web of Science, Scopus, Cochrane, PsycINFO and Web of Science were searched. Medical subject headings (MeSH) and free text used for the search were as follows: “traditional postnatal practice” or “postnatal confinement” or “postnatal tradition” or “postnatal ritual” or “traditional postpartum practice” or “postpartum confinement” or “postpartum tradition” or “postpartum ritual” or “doing-the-month” or “zuo-yue-zi”, and “depression”, and “Chinese” or “China”.

Participant or population: Chinese women population.

Intervention: The intervention is traditional postpartum confinement practice "doing-the-month".

Comparator: Not applicable.

Study designs to be included: Cross-sectional studies and randomized controlled studies.

Eligibility criteria: Studies were included if they were published English. Studies of female human participants were included. In-vivo, in-vitro, in-silico and animal studies were excluded. Meta-analysis, reviews, qualitative studies, case reports, studies with only abstracts or protocols available were excluded.

Information sources: PubMed, Embase, Web of Science, Scopus, Cochrane, PsycINFO and Web of Science between the time range of 2000-2022.

Main outcome(s): Assessment instrument for depression, postnatal depression status and the strength of association.

Quality assessment / Risk of bias analysis: A quality of assessment was conducted using the JBI critical appraisal tool was implemented to evaluate the cross-sectional studies and randomized controlled trials selected.

Strategy of data synthesis: The data are systhesized by either the protective effects, harmful effects or non-conclusive effects of "doing-the-month" on postnatal depression.

Subgroup analysis: Not applicable.

Sensitivity analysis: Not applicable.

Language restriction: Only English literatures were included.

Country(ies) involved: China.

Yang et al. Inplasy protocol 202320102. doi:10.37766/inplasy2023.2.0102 Downloaded from https://inplasy.com/inplasy-2023-2-0102/

Keywords: Maternal health; Postpartum depression; Postnatal care; Chinese; Women.

Contributions of each author:

Author 1 - Xiao Yang - Author 1 drafted the manuscript, performed the database searching, title and abstract screening and full-text assessment.
Email: xyang97@jh.edu
Author 2 - Mujie Qiu - Author 2 performed the database searching, title and abstract screening and full-text assessment.
Author 3 - Yichun Yang - The author contributed to data extraction and quality assessment of the systematic review.
Author 4 - Kun Tang - The author was invited for discussion and decision if there were inconsistent opinions between the two reviewers, provided feedback and approved the final manuscript.

Yang et al. Inplasy protocol 202320102. doi:10.37766/inplasy2023.2.0102 Downloaded from https://inplasy.com/inplasy-2023-2-0102/
